# *catena*-Poly[heptyl­enedi­ammonium [[tetra­chloridobismuthate(III)]-μ-chlorido]]

**DOI:** 10.1107/S1600536813018102

**Published:** 2013-07-06

**Authors:** Ali Ouasri, Ali Rhandour, Mohamed Saadi, Lahcen El Ammari

**Affiliations:** a Département de Physique-Chimie, Laboratoire de Chimie, Centre Régional des Métiers de l’Education et de la Formation, Souissi Rabat, Morocco; b Equipe de Physico-Chimie des Matériaux Inorganiques, Université Ibn Tofail, Faculté des Sciences, BP 133, 14000 Kénitra, Morocco; c Laboratoire de Chimie du Solide Appliquée, Faculté des Sciences, Université Mohammed V-Agdal, Avenue Ibn Battouta, BP 1014, Rabat, Morocco

## Abstract

The title organic-inorganic hybrid compound, {(C_7_H_20_N_2_)[BiCl_5_]}_*n*_, consists of distorted corner-joined [BiCl_6_] octa­hedra forming zigzag polymeric anionic chains parallel to [001], separated by columns of heptyl­enedi­ammonium cations. The asymmetric unit contains two crystallographically independent bis­muth metal atoms, one of which lies on an inversion centre and the other on a twofold axis. In the crystal, the polymeric chains and cations are linked by N—H⋯Cl hydrogen bonds, forming undulating layers parallel to (110).

## Related literature

For potential applications of alkyl­ammonium halogenido­antim­onates and -bismuthates, see: Ciapala *et al.* (1997 ▶); Bednarska-Bolek *et al.* (2000 ▶); Bator *et al.* (1998 ▶). For the structures of related compounds see: Ouasri *et al.* (2001 ▶, 2012 ▶); Jeghnou *et al.* (2005 ▶); Rhandour *et al.* (2011 ▶).
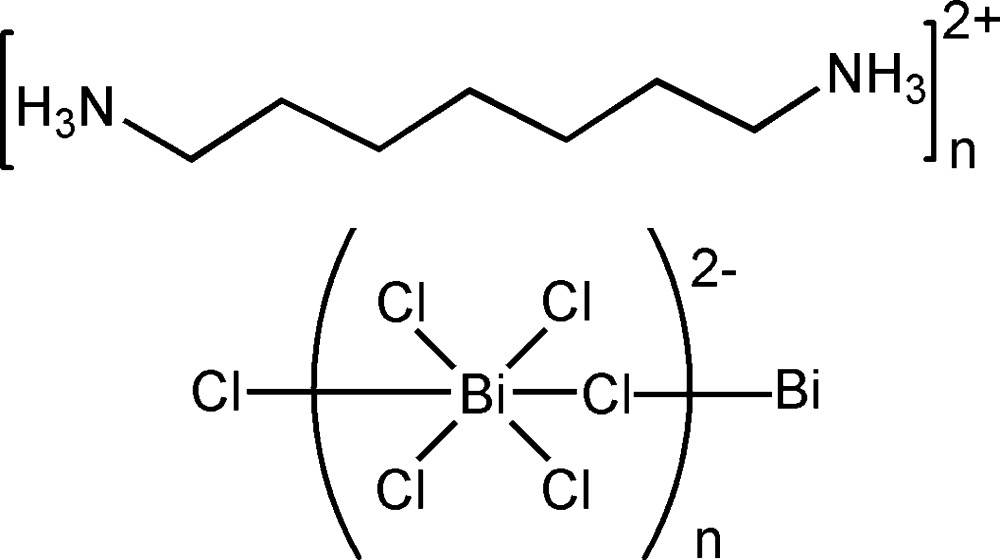


## Experimental

### 

#### Crystal data

(C_7_H_20_N_2_)[BiCl_5_]
*M* *_r_* = 518.48
Orthorhombic, 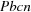

*a* = 12.2451 (5) Å
*b* = 16.5509 (6) Å
*c* = 15.8934 (6) Å
*V* = 3221.1 (2) Å^3^
*Z* = 8
Mo *K*α radiation
μ = 11.75 mm^−1^
*T* = 296 K
0.36 × 0.31 × 0.27 mm

#### Data collection

Bruker X8 APEX Diffractometer
Absorption correction: multi-scan (*SADABS*; Bruker, 2009 ▶) *T*_min_ = 0.512, *T*_max_ = 0.640
26890 measured reflections
3559 independent reflections
2700 reflections with *I* > 2σ(*I*)
*R*_int_ = 0.034

#### Refinement

*R*[*F* ^2^ > 2σ(*F* ^2^)] = 0.021
*wR*(*F* ^2^) = 0.051
*S* = 1.05
3559 reflections
138 parameters
H-atom parameters constrained
Δρ_max_ = 0.70 e Å^−3^
Δρ_min_ = −0.96 e Å^−3^

### 

Data collection: *APEX2* (Bruker, 2009 ▶); cell refinement: *SAINT* (Bruker, 2009 ▶); data reduction: *SAINT*; program(s) used to solve structure: *SHELXS97* (Sheldrick, 2008 ▶); program(s) used to refine structure: *SHELXL97* (Sheldrick, 2008 ▶); molecular graphics: *ORTEP-3 for Windows* (Farrugia, 2012 ▶); software used to prepare material for publication: *PLATON* (Spek, 2009 ▶) and *publCIF* (Westrip, 2010 ▶).

## Supplementary Material

Crystal structure: contains datablock(s) I. DOI: 10.1107/S1600536813018102/rz5076sup1.cif

Structure factors: contains datablock(s) I. DOI: 10.1107/S1600536813018102/rz5076Isup2.hkl

Additional supplementary materials:  crystallographic information; 3D view; checkCIF report

## Figures and Tables

**Table 1:** Hydrogen-bond geometry (Å, °)

*D*—H⋯*A*	*D*—H	H⋯*A*	*D*⋯*A*	*D*—H⋯*A*
N1—H13⋯Cl2^i^	0.89	2.45	3.257 (4)	151
N2—H22⋯Cl4^ii^	0.89	2.37	3.222 (3)	159

Symmetry codes: (i) 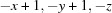; (ii) 
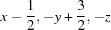
.

**Fig. 1.**
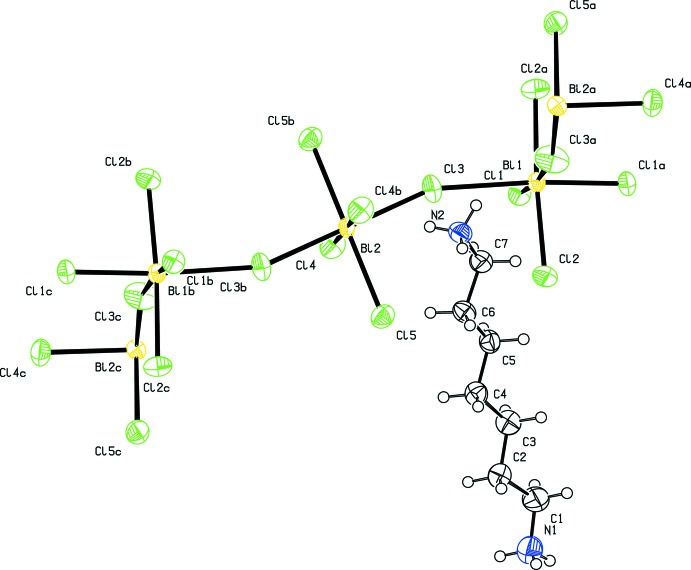
The structure of the title compound with displacement ellipsoids drawn at the 50% probability level. H atoms are represented as small circles. Symmetry codes: (a) 1-x, y, 1/2-z; (b) 1-x, 1-y, -z; (c) x, 1-y, -1/2+z.

**Fig. 2.**
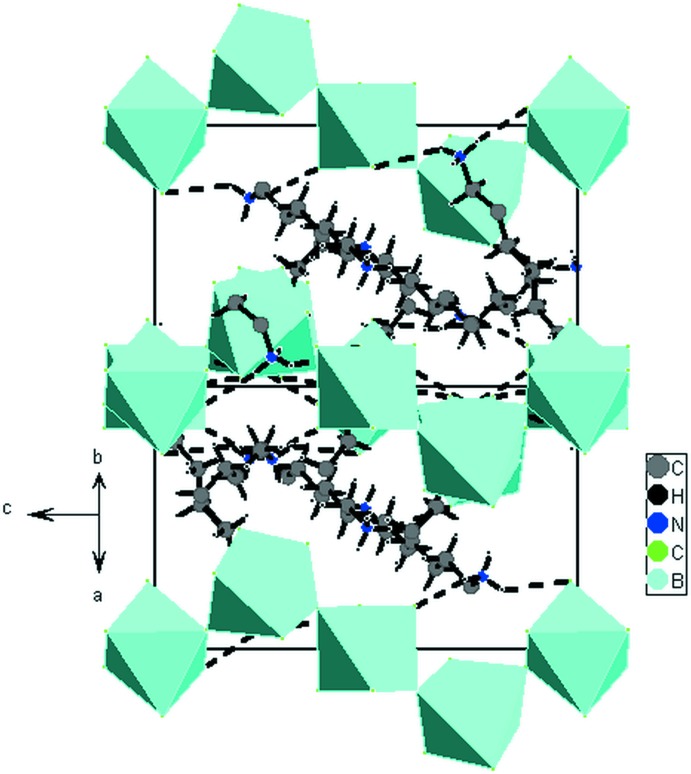
Partial plot of the title compound, showing undulated inorganic layers linked through N–H···Cl hydrogen bonds (dashed lines).

## supplementary crystallographic information

### Comment

Alkylammonium halogenoantimonates and bismuthates of general formula *R*_2_M*X*_5_, *R*_3_*M*_2_*X*_9_ and *R*_5_*M*_2_*X*_11_ (*R*: organic cations, *M*: Sb or Bi, *X*: Cl, Br or I) have recently attracted considerable attention since some of these compounds have revealed interesting properties (ferroelectric and non-linear optical) which make them promising materials from the viewpoint of applications (Ciapala *et al.*, 1997; Bednarska-Bolek *et al.*, 2000; Bator *et al.*, 1998). The crystal lattices of the halogenometallates compounds are built of distorted *MX*_6_^3-^ octahedra which are either isolated or linked to each other by corners, edges and faces. The anionic sublattices of the halogenoantimonates and bismuthates compounds of formula *R*_3_*M*_2_*X*_9_ (Ouasri *et al.*, 2001; Jeghnou *et al.*, 2005; Ouasri *et al.*, 2012; Rhandour *et al.*, 2011) are built of distorted *MX*_6_^3-^ octahedra connected with each other, by corners, edges and faces, in such a way that three halogen atoms of the coordination sphere of Sb or Bi atoms are bridging and three are terminal. The aim of the present work was to study the recently synthesized title compound by X-ray diffraction to obtain informations about its crystal structure at ambient temperature.

The structure of the organic-inorganic hybrid title compound is built up from inorganic polymeric anions and organic cations (Fig. 1). In this structure, each bismuth cation is surrounded by six chlorine anions building a distorted BiCl_6_^3-^ octahedron, with Bi—Cl distances varying from 2.5520 (8) to 2.9732 (10) Å. The octahedra are corner-joined to form one-dimensional zig-zag chains propagating along the *c* axis. The periodic length of this string is three octahedra. In the crystal structure, the chains and organic cations are linked together by N—H···Cl hydrogen bonds(Table 1) to build undulated sheets parallel to the (1 1 0) plane (Fig. 2).

### Experimental

Single crystals of the title compound were obtained by slow evaporation, at room temperature, of an aqueous solution containing stoichiometric amounts of 1,7-diaminoheptane NH_2_(CH_2_)_7_NH_2_ (acidified with HCl in a large excess) and bismuth(III) oxide Bi_2_O_3_.

### Refinement

All H atoms were located in a difference Fourier map and treated as riding, with C—H = 0.97 Å, N—H = 0.89 Å, and with *U*_iso_(H) = 1.2 *U*_eq_(C, N). One outlier (1 1 1) was omitted in the last cycles of refinement.

### Crystal data

**Table:**

(C_7_H_20_N_2_)[BiCl_5_]	*F*(000) = 1952
*M**_r_* = 518.48	*D*_x_ = 2.138 Mg m^−^^3^
Orthorhombic, *P**b**c**n*	Mo *K*α radiation, λ = 0.71073 Å
Hall symbol: -P 2n 2ab	Cell parameters from 3559 reflections
*a* = 12.2451 (5) Å	θ = 2.5–27.1°
*b* = 16.5509 (6) Å	µ = 11.75 mm^−^^1^
*c* = 15.8934 (6) Å	*T* = 296 K
*V* = 3221.1 (2) Å^3^	Block, colourless
*Z* = 8	0.36 × 0.31 × 0.27 mm

### Data collection

**Table:**

Bruker X8 APEX Diffractometer	3559 independent reflections
Radiation source: fine-focus sealed tube	2700 reflections with *I* > 2σ(*I*)
Graphite monochromator	*R*_int_ = 0.034
φ and ω scans	θ_max_ = 27.1°, θ_min_ = 2.5°
Absorption correction: multi-scan (*SADABS*; Bruker, 2009)	*h* = −15→15
*T*_min_ = 0.512, *T*_max_ = 0.640	*k* = −19→21
26890 measured reflections	*l* = −20→20

### Refinement

**Table:**

Refinement on *F*^2^	Primary atom site location: structure-invariant direct methods
Least-squares matrix: full	Secondary atom site location: difference Fourier map
*R*[*F*^2^ > 2σ(*F*^2^)] = 0.021	Hydrogen site location: inferred from neighbouring sites
*wR*(*F*^2^) = 0.051	H-atom parameters constrained
*S* = 1.05	*w* = 1/[σ^2^(*F*_o_^2^) + (0.0185*P*)^2^ + 4.1437*P*] where *P* = (*F*_o_^2^ + 2*F*_c_^2^)/3
3559 reflections	(Δ/σ)_max_ < 0.001
138 parameters	Δρ_max_ = 0.70 e Å^−^^3^
0 restraints	Δρ_min_ = −0.96 e Å^−^^3^

### Special details

**Table:**

Geometry. All s.u.'s (except the s.u. in the dihedral angle between two l.s. planes) are estimated using the full covariance matrix. The cell s.u.'s are taken into account individually in the estimation of s.u.'s in distances, angles and torsion angles; correlations between s.u.'s in cell parameters are only used when they are defined by crystal symmetry. An approximate (isotropic) treatment of cell s.u.'s is used for estimating s.u.'s involving l.s. planes.
Refinement. Refinement of *F*^2^ against all reflections. The weighted *R*-factor *wR* and goodness of fit *S* are based on *F*^2^, conventional *R*-factors *R* are based on *F*, with *F* set to zero for negative *F*^2^. The threshold expression of *F*^2^ > σ(*F*^2^) is used only for calculating *R*-factors(gt) *etc*. and is not relevant to the choice of reflections for refinement. *R*-factors based on *F*^2^ are statistically about twice as large as those based on *F*, and *R*- factors based on all data will be even larger.

### Fractional atomic coordinates and isotropic or equivalent isotropic displacement parameters (Å^2^)

**Table:**

	*x*	*y*	*z*	*U*_iso_*/*U*_eq_	
C1	0.6871 (4)	0.7642 (3)	0.0475 (3)	0.0677 (14)	
H1A	0.7050	0.7509	0.1053	0.081*	
H1B	0.6323	0.7259	0.0286	0.081*	
C2	0.6390 (3)	0.8465 (2)	0.0455 (2)	0.0451 (9)	
H2A	0.6214	0.8611	−0.0121	0.054*	
H2B	0.6916	0.8853	0.0668	0.054*	
C3	0.5365 (3)	0.8490 (2)	0.0987 (3)	0.0463 (9)	
H3A	0.4841	0.8114	0.0750	0.056*	
H3B	0.5547	0.8298	0.1547	0.056*	
C4	0.4816 (3)	0.9305 (2)	0.1073 (3)	0.0441 (9)	
H4A	0.4584	0.9489	0.0522	0.053*	
H4B	0.5339	0.9693	0.1290	0.053*	
C5	0.3835 (3)	0.9273 (2)	0.1654 (2)	0.0415 (9)	
H5A	0.3273	0.8947	0.1389	0.050*	
H5B	0.4048	0.9002	0.2169	0.050*	
C6	0.3346 (3)	1.0084 (2)	0.1881 (2)	0.0401 (9)	
H6A	0.3904	1.0421	0.2134	0.048*	
H6B	0.3094	1.0350	0.1373	0.048*	
C7	0.2416 (4)	0.9995 (2)	0.2478 (2)	0.0475 (11)	
H7A	0.1834	0.9698	0.2204	0.057*	
H7B	0.2655	0.9683	0.2960	0.057*	
N1	0.7835 (4)	0.7542 (3)	−0.0032 (2)	0.0658 (11)	
H11	0.8014	0.7021	−0.0052	0.079*	
H12	0.8382	0.7822	0.0192	0.079*	
H13	0.7704	0.7720	−0.0551	0.079*	
N2	0.1985 (3)	1.0779 (2)	0.27729 (18)	0.0453 (8)	
H21	0.1546	1.0699	0.3211	0.054*	
H22	0.1612	1.1015	0.2359	0.054*	
H23	0.2537	1.1097	0.2926	0.054*	
Cl1	0.55628 (8)	0.15799 (5)	0.13916 (5)	0.0390 (2)	
Cl2	0.29126 (8)	0.25115 (6)	0.19982 (6)	0.0504 (2)	
Cl3	0.54355 (11)	0.38672 (7)	0.12014 (7)	0.0637 (3)	
Cl4	0.54647 (9)	0.38534 (6)	−0.11631 (6)	0.0493 (2)	
Cl5	0.29132 (8)	0.45097 (6)	−0.01712 (6)	0.0465 (2)	
Bi1	0.5000	0.261494 (10)	0.2500	0.02717 (6)	
Bi2	0.5000	0.5000	0.0000	0.02820 (6)	

### Atomic displacement parameters (Å^2^)

**Table:**

	*U*^11^	*U*^22^	*U*^33^	*U*^12^	*U*^13^	*U*^23^
C1	0.073 (3)	0.062 (3)	0.068 (3)	0.024 (3)	0.022 (3)	0.008 (2)
C2	0.047 (2)	0.043 (2)	0.045 (2)	0.0008 (18)	−0.0039 (18)	−0.0019 (17)
C3	0.052 (2)	0.040 (2)	0.046 (2)	−0.0019 (19)	0.0013 (19)	−0.0077 (18)
C4	0.052 (2)	0.035 (2)	0.046 (2)	0.0019 (17)	0.0019 (18)	−0.0005 (17)
C5	0.045 (2)	0.038 (2)	0.041 (2)	−0.0006 (17)	0.0000 (17)	0.0060 (16)
C6	0.041 (2)	0.041 (2)	0.038 (2)	−0.0072 (16)	0.0024 (18)	−0.0011 (16)
C7	0.043 (2)	0.046 (3)	0.054 (3)	0.0006 (17)	0.007 (2)	0.0091 (19)
N1	0.065 (3)	0.076 (3)	0.057 (2)	0.012 (2)	−0.002 (2)	0.0057 (19)
N2	0.0392 (17)	0.061 (2)	0.0356 (15)	−0.0007 (16)	0.0033 (14)	0.0017 (15)
Cl1	0.0461 (5)	0.0372 (5)	0.0336 (4)	−0.0012 (4)	0.0044 (4)	−0.0097 (4)
Cl2	0.0391 (5)	0.0545 (6)	0.0575 (6)	0.0042 (4)	−0.0104 (5)	0.0104 (5)
Cl3	0.0720 (7)	0.0646 (8)	0.0546 (6)	−0.0119 (6)	−0.0077 (6)	0.0313 (6)
Cl4	0.0520 (6)	0.0497 (6)	0.0463 (5)	−0.0004 (5)	0.0114 (5)	−0.0126 (5)
Cl5	0.0408 (5)	0.0531 (6)	0.0457 (5)	−0.0049 (4)	0.0064 (4)	−0.0023 (4)
Bi1	0.03136 (10)	0.02362 (10)	0.02653 (9)	0.000	0.00112 (8)	0.000
Bi2	0.03338 (10)	0.02704 (11)	0.02418 (9)	−0.00266 (8)	0.00014 (8)	0.00424 (6)

### Geometric parameters (Å, º)

**Table:**

C1—N1	1.439 (6)	C7—H7A	0.9700
C1—C2	1.485 (6)	C7—H7B	0.9700
C1—H1A	0.9700	N1—H11	0.8900
C1—H1B	0.9700	N1—H12	0.8900
C2—C3	1.515 (5)	N1—H13	0.8900
C2—H2A	0.9700	N2—H21	0.8900
C2—H2B	0.9700	N2—H22	0.8900
C3—C4	1.513 (6)	N2—H23	0.8900
C3—H3A	0.9700	Cl1—Bi1	2.5520 (8)
C3—H3B	0.9700	Cl2—Bi1	2.6830 (10)
C4—C5	1.517 (5)	Cl3—Bi2	2.7287 (10)
C4—H4A	0.9700	Cl3—Bi1	2.9732 (10)
C4—H4B	0.9700	Cl4—Bi2	2.7097 (9)
C5—C6	1.512 (5)	Cl5—Bi2	2.6948 (9)
C5—H5A	0.9700	Bi1—Cl1^i^	2.5520 (8)
C5—H5B	0.9700	Bi1—Cl2^i^	2.6830 (10)
C6—C7	1.490 (5)	Bi1—Cl3^i^	2.9732 (10)
C6—H6A	0.9700	Bi2—Cl5^ii^	2.6948 (9)
C6—H6B	0.9700	Bi2—Cl4^ii^	2.7097 (9)
C7—N2	1.477 (5)	Bi2—Cl3^ii^	2.7287 (10)
			
N1—C1—C2	114.8 (4)	C1—N1—H12	109.5
N1—C1—H1A	108.6	H11—N1—H12	109.5
C2—C1—H1A	108.6	C1—N1—H13	109.5
N1—C1—H1B	108.6	H11—N1—H13	109.5
C2—C1—H1B	108.6	H12—N1—H13	109.5
H1A—C1—H1B	107.6	C7—N2—H21	109.5
C1—C2—C3	109.9 (3)	C7—N2—H22	109.5
C1—C2—H2A	109.7	H21—N2—H22	109.5
C3—C2—H2A	109.7	C7—N2—H23	109.5
C1—C2—H2B	109.7	H21—N2—H23	109.5
C3—C2—H2B	109.7	H22—N2—H23	109.5
H2A—C2—H2B	108.2	Bi2—Cl3—Bi1	158.39 (5)
C4—C3—C2	116.2 (3)	Cl1—Bi1—Cl1^i^	95.67 (4)
C4—C3—H3A	108.2	Cl1—Bi1—Cl2^i^	84.55 (3)
C2—C3—H3A	108.2	Cl1^i^—Bi1—Cl2^i^	90.53 (3)
C4—C3—H3B	108.2	Cl1—Bi1—Cl2	90.53 (3)
C2—C3—H3B	108.2	Cl1^i^—Bi1—Cl2	84.55 (3)
H3A—C3—H3B	107.4	Cl2^i^—Bi1—Cl2	172.69 (4)
C3—C4—C5	112.0 (3)	Cl1—Bi1—Cl3^i^	174.59 (3)
C3—C4—H4A	109.2	Cl1^i^—Bi1—Cl3^i^	86.58 (3)
C5—C4—H4A	109.2	Cl2^i^—Bi1—Cl3^i^	90.52 (3)
C3—C4—H4B	109.2	Cl2—Bi1—Cl3^i^	94.58 (4)
C5—C4—H4B	109.2	Cl1—Bi1—Cl3	86.58 (3)
H4A—C4—H4B	107.9	Cl1^i^—Bi1—Cl3	174.59 (3)
C6—C5—C4	115.3 (3)	Cl2^i^—Bi1—Cl3	94.58 (4)
C6—C5—H5A	108.4	Cl2—Bi1—Cl3	90.52 (3)
C4—C5—H5A	108.4	Cl3^i^—Bi1—Cl3	91.61 (5)
C6—C5—H5B	108.4	Cl5^ii^—Bi2—Cl5	180.0
C4—C5—H5B	108.4	Cl5^ii^—Bi2—Cl4^ii^	85.37 (3)
H5A—C5—H5B	107.5	Cl5—Bi2—Cl4^ii^	94.63 (3)
C7—C6—C5	111.6 (3)	Cl5^ii^—Bi2—Cl4	94.63 (3)
C7—C6—H6A	109.3	Cl5—Bi2—Cl4	85.37 (3)
C5—C6—H6A	109.3	Cl4^ii^—Bi2—Cl4	180.00 (4)
C7—C6—H6B	109.3	Cl5^ii^—Bi2—Cl3^ii^	92.81 (3)
C5—C6—H6B	109.3	Cl5—Bi2—Cl3^ii^	87.19 (3)
H6A—C6—H6B	108.0	Cl4^ii^—Bi2—Cl3^ii^	87.43 (3)
N2—C7—C6	112.9 (3)	Cl4—Bi2—Cl3^ii^	92.57 (3)
N2—C7—H7A	109.0	Cl5^ii^—Bi2—Cl3	87.19 (3)
C6—C7—H7A	109.0	Cl5—Bi2—Cl3	92.81 (3)
N2—C7—H7B	109.0	Cl4^ii^—Bi2—Cl3	92.57 (3)
C6—C7—H7B	109.0	Cl4—Bi2—Cl3	87.43 (3)
H7A—C7—H7B	107.8	Cl3^ii^—Bi2—Cl3	180.00 (4)
C1—N1—H11	109.5		

Symmetry codes: (i) −*x*+1, *y*, −*z*+1/2; (ii) −*x*+1, −*y*+1, −*z*.

### Hydrogen-bond geometry (Å, º)

**Table:**

*D*—H···*A*	*D*—H	H···*A*	*D*···*A*	*D*—H···*A*
N1—H13···Cl2^ii^	0.89	2.45	3.257 (4)	151
N2—H22···Cl4^iii^	0.89	2.37	3.222 (3)	159

Symmetry codes: (ii) −*x*+1, −*y*+1, −*z*; (iii) *x*−1/2, −*y*+3/2, −*z*.
